# *In Vivo* Resistance to Ceftolozane/Tazobactam in *Pseudomonas aeruginosa* Arising by AmpC- and Non-AmpC-Mediated Pathways

**DOI:** 10.1155/2018/9095203

**Published:** 2018-12-23

**Authors:** Erik Skoglund, Henrietta Abodakpi, Rafael Rios, Lorena Diaz, Elsa De La Cadena, An Q. Dinh, Javier Ardila, William R. Miller, Jose M. Munita, Cesar A. Arias, Vincent H. Tam, Truc T. Tran

**Affiliations:** ^1^University of Houston College of Pharmacy, Houston, TX, USA; ^2^Molecular Genetics and Antimicrobial Resistance Unit, International Center for Microbial Genomics, Universidad El Bosque, Bogotá, Colombia; ^3^Center for Antimicrobial Resistance and Microbial Genomics, UTHealth McGovern Medical School, Houston, TX, USA; ^4^Millennium Initiative for Collaborative Research on Bacterial Resistance (MICROB-R), Santiago, Chile; ^5^Grupo de Investigación en Resistencia Antimicrobiana y Epidemiología Hospitalaria – RAEH, Universidad El Bosque, Bogotá, Colombia; ^6^Division of Infectious Diseases, UTHealth McGovern Medical School, Houston, TX, USA; ^7^Genomics and Resistant Microbes Group, Facultad de Medicina Clinica Alemana, Universidad del Desarrollo, Santiago, Chile; ^8^Department of Microbiology and Molecular Genetics, UTHealth McGovern Medical School, Houston, TX, USA; ^9^Center for Infectious Diseases, University of Texas School of Public Health, Houston, TX, USA

## Abstract

Two pairs of ceftolozane/tazobactam susceptible/resistant *P. aeruginosa* were isolated from 2 patients after exposure to *β*-lactams. The genetic basis of ceftolozane/tazobactam resistance was evaluated, and *β*-lactam-resistant mechanisms were assessed by phenotypic assays. Whole genome sequencing identified mutations in AmpC including the mutation (V213A) and a deletion of 7 amino acids (P210–G216) in the Ω-loop. Phenotypic assays showed that ceftolozane/tazobactam resistance in the strain with AmpC_V213A_ variant was associated with increased *β*-lactamase hydrolysis activity. On the other hand, the deletion of 7 amino acids in the Ω-loop of AmpC did not display enhanced *β*-lactamase activity. Resistance to ceftolozane/tazobactam in *P. aeruginosa* is associated with changes in AmpC; however, the apparent loss of *β*-lactamase activity in AmpC∆7 suggests that non-AmpC mechanisms could play an important role in resistance to *β*-lactam/*β*-lactamase inhibitor combinations.

## 1. Introduction

The rise of multidrug-resistant (MDR) *Pseudomonas aeruginosa* threatens the use of available antibiotics, including carbapenems [1, 2]. The new cephalosporin/*β*-lactamase inhibitor combination ceftolozane/tazobactam (C/T) has provided a viable alternative for treatment of infections due to MDR *P. aeruginosa* [1, 2]. Indeed, many reports have confirmed the success of C/T to combat these organisms [3, 4]. Unfortunately, resistance to C/T compromises the effectiveness of this antibiotic [3, 4]. Previous reports have suggested that the mechanism of C/T resistance is associated with changes in the Ambler class C *β*-lactamase AmpC [4, 5].

In this work, 2 patients who developed C/T resistance during therapy are described and their isolates of MDR *P. aeruginosa* before (susceptible) and after (resistant) treatment with *β*-lactams were recovered. We investigated the genetic basis of C/T resistance by whole genome sequencing, and the contribution of AmpC *β*-lactamase activity to the resistance phenotype was evaluated.

## 2. Case Presentation

**Case 1 .**

A 21-year-old African American female with a history of autoimmune hemolytic anemia (receiving prednisone 5 mg orally every day) presented with acute vascular necrosis of the right femoral head after a fall. The patient had a history of polymicrobial bacteremia, including *P. aeruginosa*, which required prolonged courses of antibiotics with ceftazidime (CAZ), cefepime, meropenem, and amikacin. During her hospitalization, the patient was found to be bacteremic with *P. aeruginosa* (susceptible to aztreonam, CAZ, C/T, ceftazidime-avibactam (CZA), and polymyxin B (isolate **PA2312**)). Aztreonam 1 g intravenous (IV) every 8 hour (q8 h) was started with successful clearance after five days, and the patient was switched to CAZ 1 g IV q8 h for an additional 13 days. Four days after CAZ was discontinued, she developed a recurrent bloodstream infection due to *P. aeruginosa*. The recurrent isolate (**PA2428**) was resistant to all drugs, including aminoglycosides, C/T, and CZA, except polymyxin B (Table 1). The patient was initiated on colistin 3 mg/kg IV q12 h plus CAZ 1 g IV q8 h. After four days, therapy was transitioned to polymyxin B 15,000 units/kg IV q12 h (after 25,000 units/kg loading dose) plus C/T 1.5 g q8 h. Despite documented microbiologic clearance, the patient expired after 7 days of therapy.

**Case 2 .**

A 57-year-old man with a history of nonischemic cardiomyopathy, requiring a left ventricular assist device (LVAD), presented with drainage from the LVAD exit site. The patient had a history of recurrent LVAD exit site infections that included *Escherichia coli*, *Acinetobacter* spp., *Enterococcus*, and MDR *P. aeruginosa* (susceptible to C/T). For the last episode, in which *P. aeruginosa ***HOU2365** was isolated from the exit site, the patient received C/T for 10 weeks prior to admission. During his hospitalization, the patient underwent surgical debridement of the LVAD, and an isolate of *P. aeruginosa* was collected from the LVAD (**HOU2366**). This isolate was found to be MDR, including high-level resistance to C/T and CZA (Table 1). The patient was treated with polymyxin B 15,000 units/kg IV q12 h (after 25,000 units/kg loading dose) plus meropenem 1 g q24 h (adjusted for renal function). The patient was maintained on this combination until his orthotopic heart transplant.

## 3. Materials and Methods

### 3.1. Susceptibility Testing

Minimum inhibitory concentrations (MICs) were determined by broth microdilution as recommended by CLSI [6], except C/T, CAZ, and CZA MICs, which were determined by Etest on Mueller-Hinton agar (MHA) as recommended by the manufacturer.

### 3.2. Whole Genome Analysis

Paired-end sequence reads were generated on the Illumina MiSeq Sequencer. The software used for assembly, visualization of data, annotation, and SNP calling included CLC Genomics Workbench V.8.5.1, RAST V.2.0, MLST V.1.8, ResFinder V.2.1, BWA, SAMtools, GATK, VCF tools, and SnpEff.

### 3.3. Detection of AmpC Overproduction by Cloxacillin (CLOX)

CAZ MICs of *P. aeruginosa* were determined by Etest on MHA alone or with CLOX 1000 *µ*g/ml and incubated overnight at 37°C. A ≥ 2-fold difference in MIC of CAZ alone and CAZ tested in combination with CLOX were considered as AmpC overproduction positive as described previously [7].

### 3.4. Carbapenemase Production Testing

The CarbaNP method was performed and interpreted as defined by the Clinical and Laboratory Standards Institute (CLSI) [6].

### 3.5. Hydrolysis Activity by Nitrocefin Degradation

The enzyme activity from crude cell lysate was done using a spectrophotometric assay of nitrocefin degradation as described previously [8]. Crude cell lysates were obtained and normalized for total protein content. Nitrocefin (0.337 mM) was used as a substrate, and changes in absorbance at 486 nm were recorded as the *β*-lactam ring was degraded. PA27853 and PAVIM2 were used as negative and positive controls, respectively.

## 4. Results and Discussion

Table 1 shows the antimicrobial susceptibility profiles of 2 sets of MDR *P. aeruginosa* collected before (PA2312 and HOU2365) and after (PA2428 and HOU2366) *β*-lactam therapy. All resistant derivatives displayed resistance not only to C/T but also to CZA. This finding suggests that cross-resistance may arise between these combinations and the mechanism of C/T resistance may also impact the activity of avibactam. Importantly, C/T-resistant *P. aeruginosa* PA2428 emerged independent of C/T exposure.

C/T-resistant isolates were found to be identical to their C/T-susceptible counterparts by pulsed-field gel-electrophoresis (data not shown). Thus, the PA and HOU *P. aeruginosa* sets were further characterized by whole genome sequencing. PA2312 and PA2428 belonged to sequence type (ST) 111 while the HOU2365 and HOU2366 were ST308. Sequences of genes encoding for *β*-lactamases in the genomes of all isolates were investigated in order to determine the genetic basis of resistance to *β*-lactam/*β*-lactamase inhibitor combinations. PA2312 and PA2428 were found to have *bla*_AmpC_, *bla*_OXA-50_, *bla*_OXA-9_, and *bla*_CARB-2_, while HOU2365 and HOU2366 harbored *bla*_AmpC_ and *bla*_OXA-50_. Comparative genome sequencing revealed that all C/T-resistant isolates exhibited mutations in chromosomal *ampC* or its associated genes but not in other *bla* genes. PA2428 harbored a Val213 ⟶ Ala (V213A) substitution in the Ω-loop of AmpC compared to C/T-susceptible PA2312. HOU2366 harbored a deletion of 7 amino acids (P210-G216) (Δ7) in the Ω-loop of AmpC. Additional relevant changes are indicated in Table 2. Of note, changes in the Ω-loop have been previously shown to compromise the substrate binding site, and these changes are thought to impact both the catalytic efficiency and spectrum of substrate specificity of AmpC to *β*-lactams, particularly those with bulky side chains such as ceftolozane [9, 10]. A E247K substitution and a deletion of 19 amino acids (G229-E247), including the Δ7 present in HOU2366, have been recently shown to greatly increase C/T and CZA MICs [5]. The V213A mutation (PA2428) has been described in association with CZA resistance in *P. aeruginosa* and Δ7 (HOU2366) was also found in C/T-resistant clinical isolates [4, 11, 12]. However, their specific role in C/T resistance has not been elucidated [4, 11, 12].

As changes in AmpC were associated with C/T resistance in clinical isolates of *P. aeruginosa*, contribution of AmpC in the development of C/T resistance was evaluated. *P. aeruginosa* parental strain PA2312 was susceptible to CAZ with and without the addition of CLOX, indicating the lack of AmpC overproduction [7]. Conversely, addition of CLOX decreased the CAZ MIC of C/T-resistant PA2428 from ≥256 *µ*g/ml to 24 *µ*g/ml, consistent with an overproduction of the AmpC enzyme. Of note, AmpC overproduction in *P. aeruginosa* has been shown to be associated with changes in AmpD, present in PA2428 (Table 2), where Moyá et al. reported an increase in relative mRNA expression of *ampC* due to the V10G mutation in AmpD [13]. In the HOU *P. aeruginosa* set, AmpC *β*-lactamase overproduction was observed in HOU2365. Interestingly, C/T-resistant derivative HOU2366 did not show any evidence of AmpC overproduction, as supported by unchanged CAZ MICs in the presence of CLOX. Whole genome analysis revealed no relevant changes in AmpD for this pair. However, both strains carried a frameshift mutation in AmpD, which resulted in a truncated and aberrant protein. Thus, AmpC *β*-lactamase overproduction is a potential mechanism for C/T resistance in PA2428 but may not be the case in HOU2366.‬‬‬‬‬‬‬‬‬‬‬‬‬‬‬‬‬‬‬‬‬‬‬‬‬‬‬‬‬‬‬‬‬

**Figure 1:**
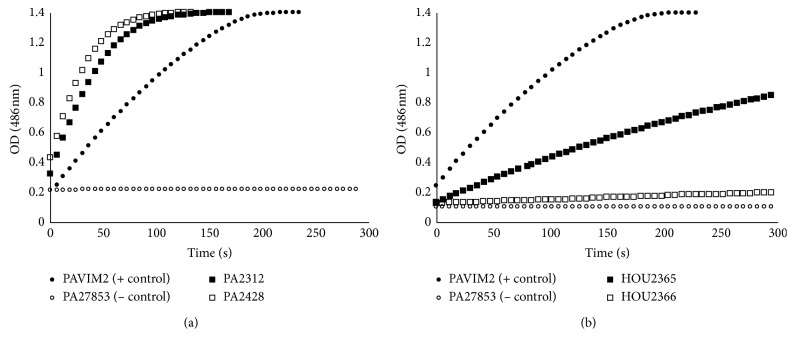
Comparative *β*-lactamase enzyme activity based on equivalent total protein.

*β*-lactamase activity of crude lysates was evaluated by spectrophotometric assay using nitrocefin as the substrate. As shown in Figure 1(a), PA2428 was able to hydrolyze nitrocefin ∼25% faster than its C/T-susceptible parent PA2312, supporting the increase in *β*-lactamase activity of AmpC as an important mediator of C/T resistance in this pair. In sharp contrast, no hydrolysis was observed in C/T-resistant derivative HOU2366 (Figure 1(b)). Instead, HOU2366 displayed a hydrolysis profile that resembled the negative control. Together, these results suggest that the likely mechanism of C/T resistance in HOU2366 may not be associated with higher *β*-lactamase hydrolysis.

Finally, to elucidate the mechanisms of carbapenem resistance in these strains, we first evaluate carbapenemase production of these strains using the CarbaNP method [6]. As shown in Table 1, all *P. aeruginosa* isolates were negative for carbapenemase production. Since repression or inactivation of the outer membrane porin OprD is a common mechanism for carbapenem resistance in *P. aeruginosa* [14], we interrogated the *oprD* gene of these isolates using whole genome data. All isolates were found to have frameshift mutations due to deletions (13-bp deletion in HOU2365/HOU2366 and 1-bp deletion in PA2312/PA2428), resulting in truncated and aberrant proteins. Collectively, these results support the lack of carbapenem susceptibility in all *P. aeruginosa* strains, regardless of C/T susceptibility.

## 5. Conclusion

In summary, the clinical cases and strain characterization presented here describe the emergence of C/T resistance after exposure to *β*-lactams. Our data suggest that C/T resistance in *P. aeruginosa* may arise due to overproduction of a mutated AmpC but it can also be independent of AmpC or *β*-lactamase activity. Further evaluation of the mechanisms of C/T resistance in *P. aeruginosa* and alternative therapies are urgently needed.

## Figures and Tables

**Table 1:** Antimicrobial susceptibility, *β*-lactamase overproduction assay, and carbapenemase production assay of *P. aeruginosa* strains.

Isolate	MIC (*µ*g/ml)^a^									CarbaNP
	FEP^b^	PTZ^b^	CZA^c^	C/T^c^	IMI^b^	MER^b^	DOR^b^	CAZ^c^		
								MH^d^	+ CLOX^d^	
PA2312	16	>128/4	4/4	2/4	32	32	64	1	1	NEG
PA2428	>32	>128/4	>64/4	64/4	32	32	16	≥256	24	NEG

HOU2365	32	>128/4	4/4	2/4	16	8	4	8	2	NEG
HOU2366	64	64/4	>64/4	>128/4	4	32	16	≥256	≥256	NEG

^a^CAZ: ceftazidime; C/T: ceftolozane/tazobactam; CZA: ceftazidime/avibactam; DOR: doripenem; FEP: cefepime; IMI: imipenem; MER: meropenem; PTZ: piperacillin/tazobactam. ^b^MIC determined by broth microdilution; ^c^MIC determined by Etest; ^d^ceftazidime (CAZ) MIC as determined by Etest in the presence/absence of cloxacillin (CLOX) 1000 *µ*g/ml.

**Table 2:** Summary of amino acid changes in C/T-resistant compared to C/T-susceptible *P. aeruginosa*.

Strain	Predicted gene product	Predicted amino acid change	Comments
PA2428^a^	AmpD	Val10 ⟶ Gly	*N*-Acetylmuramoyl-L-alanine amidase
	AmpC	Val213 ⟶ Ala	*β*-Lactamase
	DacB	Gly87 ⟶ Asp	D-Alanyl-D-alanine carboxypeptidase

HOU2366^b^	MexR	Lys67 ⟶ Glu	Transcriptional regulator
	AmpC	Δ21 bp 210-216	*β*-Lactamase
	DUF	Asp265 ⟶ Ala	Predicted signal transduction protein
	PuuA	Leu236 ⟶ Pro	Gamma-glutamyl-putrescine synthetase

^a^Compared to C/T-susceptible PA2312; ^b^compared to C/T-susceptible HOU2365.
