# Corrigendum to “*In Vivo* Resistance to Ceftolozane/Tazobactam in *Pseudomonas aeruginosa* Arising by AmpC- and Non-AmpC-Mediated Pathways”

**DOI:** 10.1155/2019/8314349

**Published:** 2019-09-02

**Authors:** Erik Skoglund, Henrietta Abodakpi, Rafael Rios, Lorena Diaz, Elsa De La Cadena, An Q. Dinh, Javier Ardila, William R. Miller, Jose M. Munita, Cesar A. Arias, Vincent H. Tam, Truc T. Tran

**Affiliations:** ^1^University of Houston College of Pharmacy, Houston, TX, USA; ^2^Molecular Genetics and Antimicrobial Resistance Unit, International Center for Microbial Genomics, Universidad El Bosque, Bogotá, Colombia; ^3^Center for Antimicrobial Resistance and Microbial Genomics, UTHealth McGovern Medical School, Houston, TX, USA; ^4^Millennium Initiative for Collaborative Research on Bacterial Resistance (MICROB-R), Santiago, Chile; ^5^Grupo de Investigación en Resistencia Antimicrobiana y Epidemiología Hospitalaria–RAEH, Universidad El Bosque, Bogotá, Colombia; ^6^Division of Infectious Diseases, UTHealth McGovern Medical School, Houston, TX, USA; ^7^Genomics and Resistant Microbes Group, Facultad de Medicina Clinica Alemana, Universidad del Desarrollo, Santiago, Chile; ^8^Department of Microbiology and Molecular Genetics, UTHealth McGovern Medical School, Houston, TX, USA; ^9^Center for Infectious Diseases, University of Texas School of Public Health, Houston, TX, USA

In the article titled “*In Vivo* Resistance to Ceftolozane/Tazobactam in *Pseudomonas aeruginosa* Arising by AmpC- and Non-AmpC-Mediated Pathways” [1], a grant number was missing. The Acknowledgments section should be updated as follows.

We thank Morouge Alramadhan for technical support. We also thank the Universidad El Bosque for financial support for whole genome sequencing (Grant no. PCI-2017-9447). This work was supported by the National Institutes of Health (Grant nos. K08-AI113317 (TTT) and K24-AI121296 (CAA)), the Millennium Science Initiative of the Ministry of Economy, Development and Tourism, Government of Chile (JMM), and the Comision Nacional de Investigacion Cientifica y Tecnologica (CONICYT), FONDECYT 1171805, Ministry of Education, Government of Chile (JMM).
